# Understanding Patients' Experiences of Digital Ethics in a Digitalised Healthcare System

**DOI:** 10.1002/nop2.70736

**Published:** 2026-09-04

**Authors:** Aysun Bayram, Betül Bal, Alvisa Palese

**Affiliations:** ^1^ Department of Nursing Fundamentals, Faculty of Health Sciences Karadeniz Technical University Trabzon Turkey; ^2^ Department of Nursing, Akdağmadeni School of Health Yozgat Bozok University Yozgat Turkey; ^3^ Department of Medical Science University of Udine Udine Italy

**Keywords:** digital ethics, digital health, ethics, health technology, patient, qualitative study

## Abstract

**Objective:**

To explore patients' experiences and perceptions of using digital health technologies, and their ethical evaluations regarding the processing of personal health data in healthcare digital environments.

**Methods:**

An interpretive phenomenological qualitative study reported in accordance with the Consolidated Criteria for Reporting Qualitative Research guidelines. Thirteen outpatients admitted to units at a university hospital were involved through purposive sampling. Face‐to‐face interviews were conducted using a semi‐structured guide. Data were analysed using a thematic analysis approach.

**Results:**

Patients' experiences with digital technologies are multidimensional, with convenience and limitations coexisting. Privacy protection, autonomy over health data, trust in the system and healthcare professionals, data security, and procedural fairness are experienced as compromised. These ethical breaches were associated with patients' perceptions of negative emotional responses and power loss. Participants described themselves as passive users of the systems, particularly due to a lack of transparency in data access and limited opportunities to correct, delete, or restrict the sharing of their data. Strengthening informed consent processes and digital literacy initiatives that enable patients to make informed use of digital systems are fundamental components of ethical protection.

**Conclusions:**

While digital health technologies offer functional conveniences for patients, they present significant ethical vulnerabilities. Privacy violations, weakening of data autonomy, and erosion of trust fundamentally affect data security and the relationship patients have with digital health systems and the wider healthcare system. The rejection of being passive victims in these processes clearly reveals the need for patient‐centred digital health governance that prioritises transparent, traceable, and accountable systems, along with data controllability.

**Implications for Nursing Practice:**

This study shows that nurses are essential in promoting patients' ethical and informed use of digital health systems. Nurses should foster patients' digital health literacy, inform them of their rights related to personal health data and consent, identify and report ethical concerns in digital health systems, and encourage patients' active involvement in decisions about their health data. These practices help empower patient autonomy, build trust in digital health systems, and support the delivery of ethical digital health services.

**Patient or Public Contribution:**

Patients who utilised digital health systems participated in this study exclusively as research subjects. To enhance the credibility of the findings, member checking was conducted with two participants, and their feedback on the emerging themes was obtained. Neither patients nor members of the public were involved in the study's design, conduct, data analysis, interpretation of results, or dissemination planning.

## 1 Introduction

Comprehensive digital health infrastructures have been developed in many countries worldwide (e.g., Finland, Germany, and Türkiye), where personal health data are managed through national digital platforms (Slawomirski et al. 2023). Türkiye is among the countries where national digital health applications such as e‐Nabız, e‐Reçete, and the Central Physician Appointment System (MHRS) are widely used and where digital health transformation has become a core components of health policies. Developed within this framework, the e‐Nabız Personal Health System has become a key component of patient‐centered healthcare by providing individuals with online access to their laboratory results, prescriptions, imaging records, and other personal health data (Birinci 2023). These systems aim to increase patients' access to health information, support their self‐management capacities, and contribute to care quality and patient safety by strengthening patient–healthcare professional interaction (Birinci 2023; Slawomirski et al. 2023; World Health Organization 2021a). Furthermore, the realisation of the expected benefits of digital health systems is closely linked to how patients experience these systems, how much they trust them, and their perceptions of the management of their personal health data in a digital environment (Agarwal et al. 2010; Brands et al. 2022; Ienca et al. 2021; Walker et al. 2017). The growing adoption of digital health applications in Türkiye makes it crucial to investigate patient experiences and the accompanying ethical considerations, both for national health policies and for the development of patient‐centered digital health services.

The World Health Organisation (WHO), while highlighting the potential of digital health systems to increase access to, continuity of, and effectiveness of health services, also notes that these technologies pose significant ethical risks (World Health Organization 2016). WHO reports state that digital health applications may pose ethical issues, including privacy and security of personal health data, data ownership and secondary data use, algorithmic biases, lack of transparency and accountability, weakening of informed consent processes, and deepening of the digital divide (World Health Organization 2016, 2019, 2021b). Qualitative studies conducted in recent years show that digital health technologies are evaluated not only based on their technical performance or usability but also within the framework of users' expectations regarding trust, privacy, autonomy, transparency, and patient‐centred care (Ienca et al. 2021; Jongsma et al. 2021). In particular, the disregard of ethical principles can directly affect patients' trust in, acceptance of, and experience with digital health technologies. In this regard, studies examining the relationship between the ethical dimension and patient experience reveal that values such as justice, autonomy, privacy, security, responsibility, and procedural values (transparency, accountability, and inclusivity) in digital health applications are closely linked to patients' perceptions and experiences (Brall et al. 2019; Buhr et al. 2025). The risk of privacy violations, unauthorised access, insufficient user information, and policy uncertainties regarding data integrity and protection deepen patients' ethical concerns about digital health systems and weaken trust (Algarni 2019; Bani Issa et al. 2020; Iwaya et al. 2020). A recent review also shows that patients' trust in digital health applications depends on who has access to their personal health data, the purposes for which the data is used, and the extent to which these processes are conducted transparently (Kassam et al. 2023). In a participatory qualitative study conducted in the context of electronic health records, artificial intelligence, robotics, and smart health technologies, Minartz et al. (2025) found that perceptions of security are influenced by factors such as data protection, a sense of control, trust, user support, and technology design. These findings demonstrate that ethical principles and patient experience in digital health systems are not independent of one another but mutually shape one another. In this context, perceptions regarding who has control over data in digital systems, within what boundaries and by whom data are accessible, as well as the potential effects of incorrect or incomplete information on care processes, directly affect their healthcare participation behaviours. Therefore, these ethics‐ and security‐based vulnerabilities necessitate that such technologies be addressed not merely as technical infrastructures, but also in line with patients' experiences, expectations, and ethical assessments. This approach is of critical importance for the development of patient‐centred digital health services and the strengthening of ethical governance.

The current literature predominantly examines patients' experiences with digital health technologies (Gybel Jensen et al. 2024), their satisfaction levels (Houser et al. 2023; Kurşun and Kaygısız 2018; Rony et al. 2024), the usability and acceptability of systems (Brands et al. 2022), their accessibility, effects on self‐efficacy and self‐management (Farmer et al. 2017), and the benefits they provide (Kruse et al. 2021; Wechkunanukul et al. 2020). A review study also reports that the key factors facilitating the adoption of digital health technologies are empowerment, self‐management, and personalization; conversely, digital health literacy, health literacy, and privacy concerns pose significant barriers (Madanian et al. 2023). Furthermore, the same study emphasises a significant gap between the development of digital health technologies and users' actual experiences, and highlights the need for research that centers the patient's perspective (Madanian et al. 2023). In this regard, qualitative studies published in recent years show that patients evaluate digital health technologies not only in terms of functionality but also in terms of values such as trust, privacy, autonomy, transparency, and ethical acceptability. For example, Ienca et al. demonstrated that when older adults evaluate digital health interventions, ethical concerns, data privacy, perceptions of autonomy, and trust are integral components of their experience with technology use. Similarly, Jongsma et al. (Jongsma et al. 2021) reported that digital health applications reshape the patient‐physician relationship and influence perceptions of trust, shared decision‐making, and ethical responsibility. In a more recent study, Minartz et al. (2025) found that individuals' perception of “feeling safe” is one of the key determinants of the adoption of digital health technologies, and that a sense of control, data security, and human support reinforce this perception. In addition, the vast majority of existing studies have focused on specific patient groups (e.g., older adults), specific clinical areas (e.g., obstetric care), or specific digital health applications. Studies that holistically address patients' perceptions of digital health systems, their experiences using them, and their ethical evaluations within the context of a comprehensive digital health infrastructure widely used nationally are quite limited. Particularly in countries like Türkiye that are implementing digital health transformation at the national level, there is a lack of qualitative evidence regarding how patients interpret these systems and evaluate them from an ethical perspective. Moreover, it remains unclear how ethical deliberation regarding the processing of personal health data in digital environments interacts with the patient experience in the context of data privacy, access authorization, data governance, transparency, and the limits of data sharing (Jongsma et al. 2021; Kassam et al. 2023). Patient‐Reported Experience Measures (PREMs) are standardised instruments that enable evaluation of healthcare services from the user experience perspective and contribute to the development of person‐centered healthcare systems (Fujisawa and Klazinga 2017; Organisation for Economic Co‐operation and Development ‐OECD 2025). The OECD PaRIS report (Organisation for Economic Co‐operation and Development‐OECD 2026), meanwhile, demonstrates that technical infrastructure alone does not ensure meaningful patient engagement in digital health systems; rather, information continuity, access to health records, and the patient experience are closely linked to trust, patient‐centredness, and perceived quality of care (Organisation for Economic Co‐operation and Development‐OECD 2026). Particularly in countries where digital transformation is proceeding rapidly, the generation of evidence based on user experience is of critical importance for the development of health services. Accordingly, the aim of this study is to reveal patients' perceptions of digital health systems, their usage experiences, and their ethical evaluations regarding the processing of personal health data in digital environments. This study contributes to the existing literature by taking a holistic approach to the patient experience with digital health technologies, including its ethical dimensions, within the context of the national digital health system.

## 2 Methods

### 2.1 Study Design

The study employed an interpretive phenomenological qualitative design. According to Creswell and Poth (Creswell and Poth 2018), the phenomenological approach aims to provide a comprehensive understanding of participants' experiences with a particular circumstance or phenomenon, as well as their interpretations of those experiences. This method enabled a thorough analysis of patients' perceptions and experiences with digital health systems, and their ethical assessments of the processing of personal health data in digital settings. The “Consolidated Criteria for Reporting Qualitative Research (COREQ)” checklist was followed in reporting the study (Tong et al. 2007; Table S1).

### 2.2 Participants

A purposive sampling (Creswell and Poth 2018) method was used. Outpatients admitted to units at a university hospital in the Eastern Black Sea Region of Türkiye were the target participants. This approach was based on the principle that outpatients typically have a stable general clinical condition, may have fewer concerns regarding clinical issues, and perceive research involvement as less intrusive than when they are hospitalised. These prerequisites may increase their availability to participate in interviews about their experiences in the healthcare process. Furthermore, identifying outpatients fostered a more ethically secure interview process and a more feasible, sustainable data collection process.

Outpatients included in the study were aged 18 years or older and (i) had used national digital health systems at least once to receive healthcare (such as e‐Nabız or MHRS, which are used for activities like scheduling appointments online, accessing lab results, or viewing prescriptions or health records); (ii) had their own active national digital health system account (e.g., MHRS, e‐Nabız); (iii) had experience accessing or viewing their own personal health data through digital health systems, or using that data when receiving healthcare; (iv) did not have any acute physical or psychiatric health issues that would prevent them from participating in the interview, were in a stable clinical condition and receiving outpatient treatment, and had sufficient cognitive capacity to participate in the interview; (v) had proficiency in speaking and understanding Turkish; and (vi) were willing to participate in the study.

Exclusion criteria were: (i) not having had contact with digital health systems during the healthcare process; (ii) having conditions affecting participation (e.g., confusion, cognitive impairment, or a clinically critical condition); (iii) being unable to provide informed consent due to severe pain, acute stress, or psychological crisis; or (iv) having hearing, speech, or cognitive impairments that prevent communication. Although the inclusion criterion required that participants had used digital health applications or systems at least once, all participants were familiar with and used digital systems for health services. They had experience using digital health services—such as booking appointments online, accessing lab and scan results, viewing prescriptions, and reviewing personal health records—primarily through e‐Nabız and MHRS. In this respect, the participants had extensive experience aligned with the study's purpose.

Two researchers (AB, BB) assessed the interviews simultaneously, and the data collection process concluded when it was determined that new interviews were no longer generating new themes or units of meaning related to understanding the phenomenon, but were instead deepening existing themes. This decision was discussed during evaluation meetings among the researchers and was reached by mutual agreement after consulting the methodological opinion of a senior researcher (AP) experienced in qualitative research (Bradshaw et al. 2017). In phenomenological research, sample adequacy is based not only on the concept of data saturation but also on understanding the phenomenon with sufficient depth and richness (Sarfo et al. 2021). In this study, the data collection process was ended once it was determined that the phenomenon had been comprehensively understood and that further interviews would not yield meaningful new insights.

### 2.3 Data Collection

This study used a patient information form and a semi‐structured interview form (Table 1) as data collection tools. These forms were developed by the researchers based on a comprehensive literature review (Agarwal et al. 2010; Brall et al. 2019; Farmer et al. 2017; Kruse et al. 2021), and their content validity was assessed by the research team. The semi‐structured interview form was pilot‐tested with a patient who was not included in the study sample to evaluate the clarity, flow, and feasibility of the questions. Following the pilot interview, no changes were made to the content of the questions; only the wording of some questions and the interview order were adjusted to make them clearer and more coherent. This pilot interview was not included in the study. Thus, the final versions of the data collection tools were established.

**TABLE 1:** The questions of semi‐structured interview form.

No	Questions
1	What are your general thoughts on digital health record systems (e.g., e‐Nabız, MHRS, telehealth) used when receiving healthcare services?
2	What are the advantages and disadvantages you experience using digital health record systems?
3	Do you receive any support or assistance in using these systems? If so, from whom, and do you share your health information with this person?
4	Do you consult or share your health information by accessing the internet (e.g., search engines, forums, applications)? Can you explain?
5	Do you have any knowledge of how your health information is processed or shared digitally when using these systems? Can you explain?
6	Do you think you have a say in the information contained in the digital health record system?
7	Do you think your personal health data is safe in the digital health record system? Why?
8	Does the possibility of your data being shared without your permission worry you? If you have had such an experience, how did you feel?
9	Do you think your privacy is sufficiently protected in these systems? Have you experienced an incident where you felt your privacy was violated in digital health services? Can you describe it?
10	Do you think storing/keeping your health information digitally is fair and ethical? Why?
11	Do you think there should be limits on healthcare professionals' access to your digital records?
12	What types of support or solutions would be important to you if you experienced a problem or ethical violation in the digital environment?
13	What measures should be taken to build greater trust with patients in terms of digital ethics?

*Note:* e‐Nabız is a personal health information system developed by the Ministry of Health, which allows individuals to view their health information electronically in Türkiye.

Abbreviations: MHRS, Merkezi Hekim Randevu Sistemi (Central Physician Appointment System); No, Number.

The Patient Information Form includes eight demographic questions regarding age, gender, educational level, profession, presence of chronic illness, years of using digital health systems, which digital health platforms are used, and the purposes for which such technologies are used. The Semi‐Structured Interview Form consists of 13 open‐ended questions. These questions address thematic areas such as general views on the use of digital health systems, ease and difficulties experienced during use, support‐seeking practices, sharing health information in a digital environment, awareness of data processing and sharing, perceptions of data security and privacy, concerns about unauthorised data sharing, ethical evaluations, and expectations in the event of potential ethical violations.

Face‐to‐face semi‐structured interviews were used for data collection. One researcher (AB) conducted the interviews in a quiet, private room within the units in February 2026 to minimise external influences. The researcher involved in the data collection phase holds a doctoral degree in nursing (AB). Before each interview, she informed patients about the scope of the study, the data collection forms and expected response time, the confidentiality of personal information, that the data would not be used outside the scope of the research, and that they could withdraw from the study at any time without providing any justification. Verbal and written informed consent were obtained from the participants. During the interviews, the researcher encouraged participants to focus on their experiences and ensured that they felt comfortable and unrestricted while sharing their views. She kept a reflective journal to record personal thoughts and possible biases for each interview. With participants' permission, the interviews were audio‐recorded, and the recordings were stored on a password‐protected computer accessible only to the researchers. Each semi‐structured interview lasted an average of 40–45 min.

### 2.4 Data Analysis

This study used a phenomenological approach, with the analysis primarily focused on uncovering participants' lived experiences with digital health systems and the meanings they ascribed to those experiences. Accordingly, Braun and Clarke's six‐stage reflexive thematic analysis (RTA) approach was employed (Braun and Clarke 2022). Although RTA is not a method specific to phenomenology, it was chosen because it can systematically and flexibly reveal common patterns of meaning in lived experiences. Throughout the analysis, each participant's experience was first evaluated within its own context and then shared patterns of meaning were identified across participants. In this way, the phenomenological focus on lived experience was maintained throughout the analysis. The first stage of the analysis process was:

#### 2.4.1 Familiarisation With the Data

Audio recordings were listened to and transcribed verbatim by two researchers (AB, BB). The researchers read the entire transcript multiple times to become familiar with the data, noting prominent initial impressions, noteworthy concepts, and recurring meanings in participants' statements. At this stage, the researchers took reflexive notes, consistently bearing in mind that their own preconceptions might influence their interpretation of the data and focused on understanding the participants' lived experiences within their own contexts.

#### 2.4.2 Generating Initial Codes

The dataset was systematically coded by dividing it into meaningful data units aligned with the research questions by two researchers (AB, BB). The coding process was conducted inductively; codes were developed from statements reflecting the participants' lived experiences, without relying on predetermined theoretical categories. For example, statements such as “I do not know who has seen my data (P2)” and “I cannot trust the system (P6)” were grouped under codes for uncertainty about data access and distrust in digital systems.

#### 2.4.3 Searching for Themes

Similar codes were grouped under categories to create higher‐level semantic patterns and identify potential themes by two researchers (AB, BB). At this stage, while examining the relationships between codes, particular attention was paid to ensuring that the themes reflected the essence of the participants' lived experiences and the meanings they ascribed to those experiences.

#### 2.4.4 Reviewing Themes

The themes created were re‐evaluated in terms of both their internal consistency and their compatibility with the entire dataset by the same researchers. Some themes were combined, some separated, and some renamed. During this process, each theme was systematically reviewed to determine whether it meaningfully and comprehensively represented the participants' lived experiences. In cases where themes did not sufficiently align with the participants' narratives, the thematic structure was reorganised, and the review process continued until consensus was reached among the researchers.

#### 2.4.5 Defining and Naming Themes

The final themes were defined in a clear, distinctive, and analytical manner. The semantic field encompassed by each theme was clarified, and the relationships of the themes to digital health systems, data security, privacy, and ethical perceptions were explained. Sub‐themes were identified, and the thematic structure was detailed by two researchers (AB, BB).

#### 2.4.6 Producing the Report

In the final stage of the analysis, themes were reported and supported by direct quotations from participant statements. The findings were supported by direct quotes that remained faithful to the participants' lived experiences and were interpreted in light of the relevant literature. Overall, patients' ethical perceptions of digital health systems were presented in a holistic narrative.

In phenomenological research, it is important to be aware of the researcher's interpretive role. In this study, the researchers adopted a reflexive approach, mindful that their own experiences, knowledge, and assumptions could influence their interpretations throughout the data collection and analysis process. The researchers had prior knowledge and professional awareness that digital health systems could make significant contributions to healthcare, but that patients might have various concerns regarding data confidentiality, privacy, and ethical issues. Recognising that these preconceptions could influence their interpretation of the data, they continually questioned and reviewed them through reflexive evaluations throughout the analysis process. To this end, the researchers held regular analysis meetings, kept reflexive notes, and continuously discussed whether the themes accurately represented the participants' lived experiences. Quantitative data regarding the profile of patients involved were analysed using descriptive statistics (averages, frequencies, ranges). At this stage, two researchers (AB, BB) worked together, and the opinions of a third senior researcher (AP) were also obtained.

### 2.5 Trustworthiness

This study adopted Lincoln and Guba's trustworthiness framework to ensure the scientific rigour of qualitative research. Within this framework, the credibility, transferability, dependability, and confirmability of the research were systematically examined (Lincoln and Guba 1985).

#### 2.5.1 Credibility

To enhance the methodological rigour and consistency of data collection, a semi‐structured interview form was used to allow participants to describe their experiences in detail. The interviews were audio‐recorded and transcribed verbatim after each session while data collection was ongoing. This approach enabled progressive assessment of saturation (Creswell and Poth 2018). Braun and Clarke's (2022) thematic analysis approach was followed during data analysis, with repeated revisiting of the data during coding and theme development to deepen the analysis. Direct quotations from participants were included in the findings to clearly link interpretations to the data. Additionally, to assess whether the findings accurately reflected participants' experiences and to support the study's credibility, the themes were shared with two participants (P1 and P11), and member checking was conducted based on their feedback.

#### 2.5.2 Transferability

To support transferability, the study context, participant characteristics, and data collection process are described in detail. Participants' experiences using digital health systems, the clinical setting of the interviews, and the stages of the research process are clearly stated. This is intended to ensure that the findings can serve as a reference for studies in similar contexts.

#### 2.5.3 Dependability

To ensure dependability, data collection and analysis were conducted systematically and transparently. Interview questions, data collection conditions, the coding process, and theme development stages are documented in detail within the manuscript (report available from the authors). The steps followed in the thematic analysis are clearly defined to ensure traceability of the research process. During coding and theme creation, the input of a senior author (AP), an expert in qualitative research, was sought, and codes and themes were compared to strengthen consistency. Discussions continued until consensus was reached on the themes.

#### 2.5.4 Confirmability

To strengthen confirmability, the findings were developed based on the participants' narratives, and the researchers' interpretive role was evaluated using a reflexive approach throughout the analysis. Researchers (AB, BB) kept reflective notes to identify the potential impact of their assumptions and interpretations on data analysis; these notes were used to justify analytic decisions. To ensure that the researchers' interpretations were consistent with the data and transparent, the findings are supported by direct quotes from participants. Additionally, notes and decisions related to the analysis were recorded, creating a verifiable audit trail. This increased the verifiability of the findings.

## 3 Findings

A total of 13 patients participated. The mean age was 36.46 years (range 25–60), with the majority being female (10, 76.92%). A bachelor's degree was the lowest level of education, and professions ranged from software developer (1, 7.69%) to teacher (4, 30.77%). Ten participants did not have a chronic illness. The average number of years of experience using digital health systems was 8.23 (range 3–15). All participants used various technologies, with e‐Nabız and MHRS being the most popular. The primary uses of these systems were making appointments, accessing test results, and obtaining prescriptions (Table 2).

**TABLE 2:** Participants socio‐demographic characteristics and digital health system use (*N* = 13).

Characteristics	
Age, years	
Range	25–60
Mean	36.46
Gender	*n* (%)
Female	10 (76.92)
Male	3 (23.08)
Educational degree	*n* (%)
Bachelors'	9 (69.23)
Master	1 (7.69)
PhD	3 (23.08)
Profession	*n* (%)^a^
Teacher	4 (30.77)
Academician	3 (23.08)
Nurse	2 (15.38)
Retired	1 (7.69)
Software developer	1 (7.69)
Banker	1 (7.69)
Officer	1 (7.69)
Chronic illness	*n* (%)^a^
Yes	3 (23.08)
No	10 (76.92)
Using digital health systems, years	
Range	3–15
Mean	8.23
Type of digital health systems^a^	
e‐Nabız	13 (100)
MHRS	13 (100)
E‐Devlet health services module	11 (84.62)
Private health application programs (about pregnancy, menstruation, fitness, tele‐psychology)	6 (46.15)
ALO 182	8 (61.54)
Wearable health devices (smartwatch, sleep/activity tracking)	2 (15.38)
Independent/third‐party artificial intelligence tools (e.g., ChatGPT, Perplexity, etc.)	1 (7.69)
Purpose of using digital health systems^a^	*n* (%)
Accessing appointment (receiving, cancelling, changing)	13 (100)
Accessing test results	13 (100)
Obtaining prescription	13 (100)
Making diagnosis inquiry	2 (15.38)
Monitoring conditions (e.g., pregnancy)	2 (15.38)
Reviewing past examination summaries	3 (23.08)
Monitoring daily activity, heart rate and sleep	5 (38.46)
Medication reminders	2 (15.38)
Accessing dietitian follow‐up	1 (7.69)
Doctor/clinic communication/consultation	2 (15.38)

*Note:* e‐Nabız is a personal health information system developed by the Ministry of Health, which allows individuals to view their health information electronically in Türkiye. e‐Devlet is Türkiye's electronic government portal providing access to public services online. ALO 182 is the call center of the Central Physician Appointment System (MHRS), providing hospital and physician appointment services.

Abbreviations: MHRS, Merkezi Hekim Randevu Sistemi (Central Physician Appointment System); PhD, Doctor of Philosophy.

^a^ Refers to multiple responses.

Participants' experiences with digital health technologies and processes were summarised in 15 sub‐themes under four main themes: (i) Balancing Convenience and Dependency in Digital Health; (ii) Losing Control in the Digital Health Environment; (iii) When Ethical Violations Become Personal; and (iv) Building an Ethically Trustworthy Digital Health System (Table 3).

**TABLE 3:** Themes and sub‐themes.

Themes	Sub‐themes	Categorises
1. Balancing Convenience and Dependency in Digital Health	1. Ease of interaction	
	2. Barriers to use	1. Technical and systemic problems
		2. Difficulty understanding and interpreting information
		3. Personal digital inadequacies
	3. Dependency in digital health applications	1. User as a supportive agent
		2. Support dependency
2. Losing Control in the Digital Health Environment	1. Erosion of privacy	1. Uncertainty regarding data storage
		2. Unauthorised data sharing
	2. Lack of autonomy	1. Lack of knowledge regarding data rights
		2. Alienation from data rights
		3. Missing of informed consent
	3. Erosion of trust	1. System ınsecurity
		2. Loss of trust in stakeholder (e.g., healthcare professionals, government)
	4. Erosion of security	1. Unauthorised system access
		2. Unauthorised access data
		3. Unauthorised data storage
	5. Erosion of procedural values	1. Missing transparency
		2. Lack of accountability
		3. Lack of data control
3. When Ethical Violations Become Personal	1. Distress	Anger, anxiety, shame, embarrassment, loss of trust, trauma
	2. Powerlessness	—
4. Building an Ethically Trustworthy Digital Health System	1. Transparency and traceability	—
	2. Accountability	—
	3. Data controllability	—
	4. Informed consent	—
	5. Digital Literacy	—

### 3.1 Main Theme 1. Balancing Convenience and Dependency in Digital Health

This theme highlights that, although digital health systems facilitate access to healthcare, this ease of access does not always translate into an independent user experience. Technical issues, limitations in digital literacy, and the need for support have emerged as key factors shaping patients' interactions with such technologies.

#### 3.1.1 Sub‐Theme 1. Ease of Interaction

In this sub‐theme, participants identified the practicality of processes such as making appointments, accessing test and examination results, and managing personal health information as fundamental elements that facilitate interaction with the systems. Participants stated that these functions accelerate access to health services and offer a health management opportunity that is more compatible with daily life

In general, I find these systems quite practical. I can make appointments without having to go to the hospital, view my test results, and track my prescriptions. (P2)

In general, I have positive experiences; these systems make our tasks easier. For example, I can make my appointment over the phone and review my test results. Having information before going to the doctor is reassuring. Seeing the test results helps reduce my anxiety. (P3)

#### 3.1.2 Sub‐Theme 2. Barriers to Use

This sub‐theme is divided into three categories that describe the difficulties participants encountered when using digital systems. “Technical and Systemic Problems” refers to low system performance, incomplete or delayed data display, access problems, or technical failures, all of which participants described negatively as affecting user experience. “Difficulty Understanding and Interpreting Information” indicates that patients find the data in digital systems contain medical terms and that they cannot understand the information due to its dense, complex structure, making it difficult to interpret their own data. “Personal Digital Inadequacies” reports that participants' low levels of digital literacy cause difficulties in using the systems. Participants stated that they experience password recall and login problems, internet access issues in their locations, inability to create appointments in the appropriate area, and perceive the system interface as complex, especially due to low digital literacy levels.

The frequent repetition of authentication steps and the advertisements or unexpected pop‐up permission requests that appear during the process distract me and can cause me to make mistakes. (P6)

Some reports contain quite technical language. For example, when I first encountered terms like ‘placenta previa,’ I was worried because I did not know what it meant and had to wait for my doctor to get an explanation. It would be useful to have an explanatory glossary or guide for such situations. (P9)

Password procedures are quite challenging for me. Constantly changing passwords and the process of forgetting and resetting passwords becomes tiring. Also, some sites have complex interfaces; when I cannot find the information I am looking for, I experience problems during use. (P3)

### 3.2 Sub‐Theme 3. Dependency in Digital Health Applications

Participants stated that they support their close circles in using digital health applications and assume a supportive role in this process. These findings represent the “User as a Supportive Agent” category. Furthermore, some patients reported requiring assistance from others when using digital health systems, which contributed to a perceived sense of dependency. These experiences were categorised as “Support Dependency.”

I usually use the system on my own; however, I occasionally get support from my circle or provide support to my circle regarding technology‐related issues. Nevertheless, I never share my health information. (P2)

### 3.3 Main Theme 2. Losing Control in the Digital Health Environment

**FIGURE 1:**
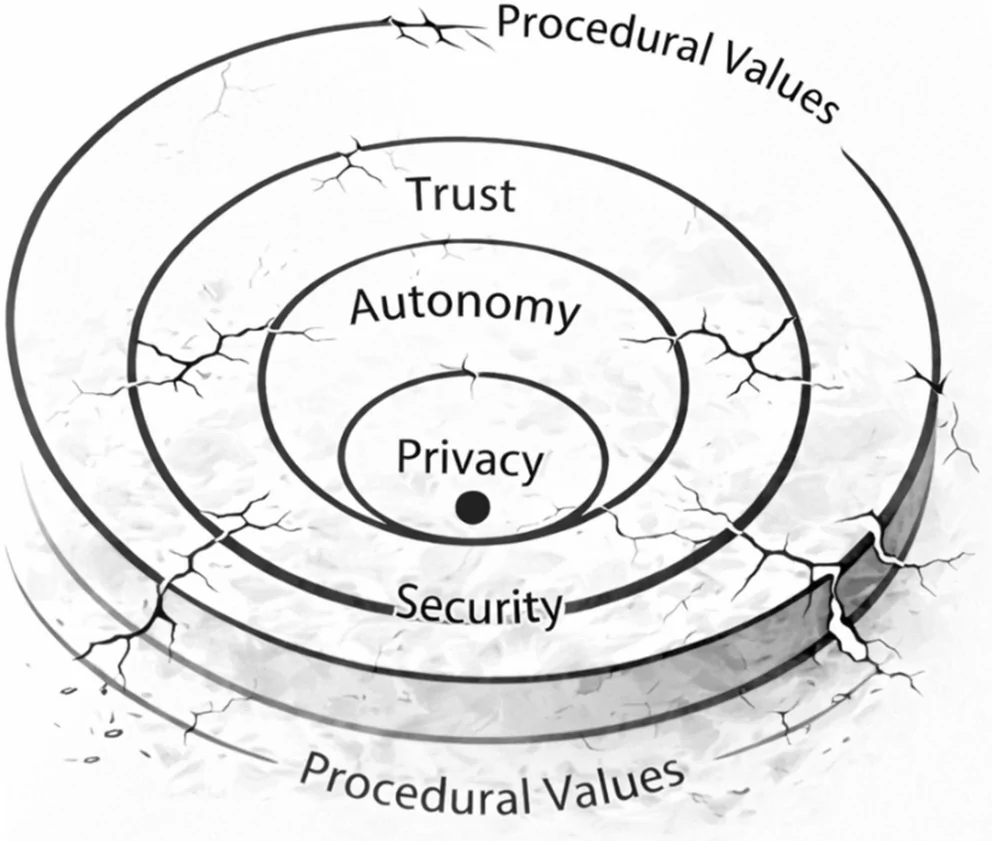
Layered ethical breaches in digital health from the patient perspective.

This theme reveals that patients interpret the ethical issues they encounter in digital health systems not as isolated incidents, but as interconnected experiences that undermine their sense of control and trust in the system. Ethical concerns related to privacy, autonomy, security, trust, and due process, along with perceived threats to these values, constitute common experiential patterns that influence participants' evaluations of these technologies. The theme consists of five subthemes (Figure 1).

#### 3.3.1 Sub‐Theme 1. Erosion of Privacy

Participants reported that privacy is insufficiently protected in digital health systems and expressed specific concerns regarding privacy. Participants expressed that they lacked information about where, how, and for how long health data are stored, resulting in “Uncertainty regarding data storage”.

I have not had a negative experience; however, the thought that data is ‘stored somewhere anyway’ bothers me. Since I know that everything on the internet can somehow be revealed, I think that privacy is not fully protected. (P4)

I do not know in detail with whom, under what conditions, and for how long the data are shared. I feel a serious uncertainty about which algorithms work on my data, how anonymisation processes are carried out, or how third‐party artificial intelligence services use this data. (P11)

Additionally, participants perceived that their own data were shared without their knowledge or consent, raising concerns about “Unauthorised data sharing”.

I think I should have a say in some matters. For example, I should be able to choose who can see my information. Right now, it feels like I do not have much control. The doctor's access is natural; however, not knowing whether a software developer or system provider accesses the data bothers me. (P4)

#### 3.3.2 Sub‐Theme 2. Lack of Autonomy

Participants stated that they were not sufficiently aware of their rights regarding their personal health data and that they lacked the authority to make decisions about the use of this data. This situation demonstrates that participants perceive their individual autonomy as constrained when interacting with digital health systems. They defined that they do not have sufficient knowledge about their legal rights regarding personal health data, suggesting a “Lack of Knowledge Regarding Data Rights”. Furthermore, participants expressed that they do not have control mechanisms such as editing, restricting access, or managing usage permissions over the data in digital health systems; therefore, they remain in a passive user position, leading to a sense of “Alienation from Data Rights”. Another identified ethical concern involves participants' experiences with the informed consent process. “Missing informed consent” reflects experiences in which participants reported their consent was not obtained regarding any rights or say about their data in digital systems.

Sometimes I feel like I have no say at all. The doctor looks at and sees my information; even if I do not want it, this information is in the system. A feeling that the control is not with me arises. (P3)

Ultimately, the information belongs to me; however, where, for how long, and by whom this data will be used is determined without asking me. For example, a test result or prescription is uploaded directly to the system and I can only view it. So the data belongs to me, but the control of the system is not with me. (P13)

I have the right to view my data; however, I do not have rights such as deleting or restricting access. Especially as a healthcare worker, I would like to know who views my information. (P10)

#### 3.3.3 Sub‐Theme 3. Erosion of Trust

Participants reported perceptions of “System Insecurity”, as the failure to provide sufficiently clear and understandable information about its functioning and concerns that health data could be misused, while technical problems experienced negatively affect the perception of trust. Participants shared perceptions that healthcare workers or stakeholders who control the systems (e.g., government, Ministry of Health) may access personal health data without justification, which was experienced by participants as a “Loss of trust in stakeholders”.

If only authorised persons have access, I do not see an ethical problem. However, since I know that systems are not completely error‐free, my ethical concerns continue. (P1)

In general, I know that the system is managed by the Ministry of Health and that data are protected with certain security protocols. However, I do not have detailed information about with whom and for what purposes the data are shared. (P12)

I do not have precise knowledge about how the systems work; however, I know that certain health institutions can access data collectively. In my own experiences, I noticed that there could be unauthorised access. This situation has caused me to seriously question the trust I have in the system. (P2)

I know that these systems are managed by the Ministry of Health; however, I do not have clear information about with whom my data are shared and which institutions can access them. I think more information should be provided on this matter. (P7)

#### 3.3.4 Sub‐Theme 4. Erosion of Security

Participants also shared a serious perception of uncertainty regarding who can access personal health data, with what authority, and for what purpose. They perceived that individuals without the appropriate rights according to their job descriptions and permissions may access health data, leading to “Unauthorised System Access”. In particular, the invisibility of access records in the systems and limited auditability reinforce this perception. Participants also expressed the belief that third parties access personal health data without explicit consent, thus reflecting “Unauthorised Access to Data”. Additionally, uncertainties regarding how and for how long data are stored, and the lack of sufficiently clear and transparent usage conditions, have further strengthened the perception of security breaches among participants, in this case leading to “Unauthorised Data Storage”.

On one occasion, I saw that another healthcare worker, other than my dietitian, accessed my past reports. At that moment, I could not understand the reason for this access and I felt uncomfortable. I think data confidentiality needs to be protected more strongly. (P8)

I think access should be ‘need‐based’. Only those responsible for the patient's care should have access to necessary information during their duty periods. Additionally, access records should be kept; who viewed what information and when should be visible to the patient. (P6)

I have experienced situations where I thought my privacy was not fully protected. For example, I noticed that my family doctor accessed my e‐Nabız system multiple times. (P1)

#### 3.3.5 Sub‐Theme 5. Erosion of Procedural Values

Participants stated that fundamental procedural values such as transparency, accountability, and data control are not adequately implemented in digital health systems. They identified “Missing Transparency” as the inability to access information about who accesses health data, for what reasons, and to what extent, as a significant ethical concern. The lack of effective complaint, feedback, and oversight mechanisms in response to ethical violations was linked by participants to a lack of clarity regarding accountability and a loss of institutional trust; this situation created a perception of a “Lack of Accountability” among them. They also expressed that their inability to perform operations such as correcting, deleting, or restricting the sharing of health data weakens their perception of control and autonomy over the data, thus experiencing “Lack of Data Control”.

I know that our data are stored in central databases and shared among public institutions with certain permissions. However, we do not fully know how these processes are supervised and who has access authority. Theoretically, I have the information, but in practice, the control is not with me. (P12)

After uploading data to the system, I have questions about how this information will be used, especially for research or third‐party analyses. I do not have a complete sense of control over the purposes for which the data are processed, whether they are anonymised or not, or how they are evaluated by companies and researchers. Therefore, although it seems as if there is a say in theory, I think these rights are not adequately protected in practice. (P11)

### 3.4 Main Theme 3. When Ethical Violations Become Personal

This theme displays that digital ethical violations extended beyond technical concerns and became deeply personal experiences, leaving patients with enduring feelings of distress and powerlessness. It comprises two sub‐themes: distress and powerlessness.

#### 3.4.1 Sub‐Theme 1. Distress

Within this sub‐theme, participants stated that the sharing of information constitutes ethical violations, exposing them to significant distress such as unease, which disrupts an individual's emotional balance. This situation has been associated with emotional reactions such as anger, anxiety, shame, embarrassment, loss of trust, and even trauma among participants.

Unauthorised sharing of health data would definitely be concerning. In such a situation, I would feel ‘exposed.’ A privacy violation means the disclosure of very personal information to the public; this would lead to feelings of shame and anger. (P4)

Unauthorised sharing of sensitive health information would create feelings of shame, embarrassment, and distrust. I have not yet experienced such a situation; however, if it had happened, I would probably feel anger, helplessness, and something similar to trauma. The use of my data beyond my control would be perceived as a violation of my personal space. (P11)

Participants emphasised that these experiences were not temporary, but rather left long‐term, permanent negative emotional effects.

On one occasion, I witnessed a patient's information being mistakenly shown to another patient's relative. The embarrassment and loss of trust experienced at that moment was quite shocking. Such a violation is not only limited to data security but also creates serious psychological trauma. (P10)

#### 3.4.2 Sub‐Theme 2. Powerlessness

Participants expressed that they felt powerless to change the way digital health systems operate or to intervene in the ethical challenges they faced. This situation has been associated by participants not only with a sense of loss of control but also a feeling of emotional powerlessness and helplessness.

On one occasion, I noticed that a technician with whom I had no direct health relationship accessed my reports in the system. I could do nothing about this situation, nothing at all. (P10)

Health information is private. Unauthorised knowledge of a disease like diabetes in the workplace or social environment can put a person in a difficult position. (P7)

### 3.5 Main Theme 4. Building an Ethically Trustworthy Digital Health System

This theme indicates that participants believe digital health systems should be restructured based on ethical principles rather than rejected outright. Participants noted that trust in such technologies can only be achieved by strengthening transparency, accountability, data control, and informed consent processes, as well as by increasing digital literacy. The theme consists of five subthemes.

#### 3.5.1 Sub‐Theme 1. Transparency and Traceability

Participants stated that having clear and traceable information about who uses health data and for what purposes would increase trust in digital health systems. They emphasised that data access records should be viewable, institutions and individuals accessing the data should be clearly visible, and transparency in data usage processes should be enhanced.

I have not personally experienced such a situation before; however, when I heard that a friend's information on e‐Nabız was accessed without permission, I was deeply shaken. After this incident, I started to follow the access permissions in the system more carefully. Digital health systems need to provide users with the ability to monitor this security as much as they need to be secure. Otherwise, the most sensitive periods of our private lives can turn into a pile of data in the hands of others; this situation is unacceptable both ethically and humanely. (P9)

Transparency should be ensured in data sharing and patients should be clearly shown who accesses which data. Additionally, two‐factor authentication, regular security tests, and user education should be implemented in the systems. Most importantly, it should be guaranteed that no data are shared without patient consent. (P12)

#### 3.5.2 Sub‐Theme 2. Accountability

Participants expressed that their trust in the systems is damaged when responsibility is unclear in cases of digital ethical violations or when feedback processes do not function; therefore, they stated that accountability is a fundamental element of patient‐centred ethical governance in digital health applications.

I think there should be an effective complaint mechanism within the system. In addition, it is important to establish an independent oversight board and to have a consultation line or patient rights unit that patients can contact. (P7)

#### 3.5.3 Sub‐Theme 3. Data Controllability

Participants stated that having limited control over data reinforces feelings of powerlessness and insecurity; conversely, the ability to correct erroneous information, see how data are processed, and update them strengthens patients' sense of autonomy.

Being able to receive quick feedback, have errors corrected promptly, and manage my own access rights in the system are very important to me. I felt uncomfortable because I was not sufficiently informed before; knowing that I have such rights from now on would be reassuring for me. I think that people who have access to the system must get permission from me, even if the system is open. I do not want some information to be seen and I think I should be asked before this data is recorded in the system, because these are my private areas. (P2)

I know that hospital data are stored in their own systems and that central systems like e‐Nabız collect this data. However, I do not have detailed information about which third parties (such as analytics companies, cloud providers, or advertising networks) this data is shared with; this situation bothers me. Since cookie policies and terms of use are usually long and technical, I cannot fully understand them and often have to check the ‘accept’ option. (P6)

#### 3.5.4 Sub‐Theme 4. Informed Consent

Participants stated that consent texts should be understandable and accessible, explicit consent should be obtained from patients in data collection processes, and additional protection mechanisms should be implemented, especially for sensitive data.

I feel that healthcare workers often see accessing information as their own right. Even if there is an access restriction, they can request the SMS code sent to my phone; however, no information is provided about why this code is requested or for what purpose it will be used. Additionally, a clear permission or consent process is not implemented. This situation creates a serious feeling of discomfort in me. (P2)

I think there is a need for clear and understandable consent forms. Instead of long and legal texts, short informative texts that explain in bullet points which data will be shared with whom and for what purpose should be preferred. (P6)

#### 3.5.5 Sub‐Theme 5. Digital Literacy

Participants emphasised that user guides for digital health applications should be provided, digital ethics training should be planned for both health professionals and the public, and digital health and digital ethics awareness should be increased through community‐based awareness programs.

Keeping health data in digital environments is a positive development for ensuring continuity in health services. However, not everyone has equal access to technology or the same level of digital literacy. This creates inequalities in digital justice, as some people do not receive equal service because they have difficulty understanding the systems:

From an ethical perspective, I think informed consent processes are not adequately implemented. (P13)

## 4 Discussion

The findings of this study reveal how patients experience and interpret digital health technologies, showing that ethical issues arise within these experiences and are a fundamental dimension shaping them. The study findings are here discussed under four main themes: (i) Balancing Convenience and Dependency in Digital Health; (ii) Losing Control in the Digital Health Environment; (iii) When Ethical Violations Become Personal; and (iv) Building an Ethically Trustworthy Digital Health System.

### 4.1 Balancing Convenience and Dependency in Digital Health

Patients' positive evaluations of digital health systems for practicality and speed indicate that they can manage their health in a way more compatible with their daily lives, supporting a patient‐centered approach (Wong et al. 2024). Time‐ and location‐independent use, instant access to health data, and the ability to manage care processes more autonomously strengthen individuals' active participation in their own health (Aytekin et al. 2025). These findings are consistent with previous studies that emphasise the conveniences provided by digital health applications for managing health information and organising service processes (Birinci 2023; Kurşun and Kaygısız 2018; Scandale 2026; Smith et al. 2020; Wong et al. 2024). Patients' reports of various barriers alongside ease of use demonstrate that these systems are not equally accessible or usable for everyone (Nebeker et al. 2021). The literature highlights that technical problems, difficulties in interpreting information, and digital literacy levels are fundamental determinants of effective use of these systems, and these findings align with the study's results (Jawad 2024; Steitz et al. 2023). Jensen et al. (Gybel Jensen et al. 2024) report that patients' experiences with digital health systems are influenced not only by the technical interface design but also by individuals' digital, cognitive, and communicative resources. This finding also shows that patients' possession of these resources creates both ease and difficulty in using the systems, thereby affecting their independence within the systems. As noted by Hjelholt et al. (2018), some individuals rely on their own social networks and resources when using and learning digital systems. In this study, most patients assumed a supportive role for others when using such technologies, while some required support themselves. The fact that the sample consisted of individuals with high levels of education and active users of digital systems may partially explain this tendency. The assumption of a supportive role can be considered positive, as it expands the use of these systems and increases other patients' learning. However, using digital health systems with support from others may pose risks to data privacy and ethical issues (Jawad 2024), as sharing health data with third parties increases the likelihood of unauthorised access and privacy violations (Bani Issa et al. 2020; Iwaya et al. 2020). The inclusion of patient relatives in digital health processes reveals hidden usage difficulties and reaches digitally hard‐to‐reach groups (Gybel Jensen et al. 2024). However, this raises ethical and governance‐related discussions, such as patient autonomy, privacy, and responsibility sharing, as it increases the risk of dependence on others in digital health systems.

### 4.2 Losing Control in the Digital Health Environment

Ethical issues in digital health applications manifest as mutually reinforcing systemic breaches rather than isolated violations. Participants' perceptions of privacy violations indicate that the principle of confidentiality, a fundamental component of ethical trust, has been compromised. Uncertainty regarding how, where, and within what boundaries health data are stored has emerged as a fundamental factor weakening trust in digital health systems. Previous studies also show that privacy and confidentiality concerns directly affect system use (Kyytsönen et al. 2024; Vayena et al. 2018). Additionally, the violation of the confidentiality of millions of health records worldwide further deepens patients' concerns (U.S. Department of Health, and Human Services, Office for Civil Rights 2026). Privacy violations are not limited to technical infrastructure problems; they must be addressed together with their organisational, environmental, and ethical dimensions (Houser et al. 2023). The perception of privacy violation deepens with patients' sense of lack of control over their data, strengthening the perception of being pushed into a passive user position in digital systems. Participants' demands for user control, selective data sharing, and transparency of access records align with the role‐based access control requirement emphasised in the literature (Pokharel and Kathayat 2025; Poulsen et al. 2025). However, although patients can view their data, the inability to control access and the ambiguity surrounding access justifications trigger a sense of ethical alienation. The combined loss of privacy and autonomy develops into a broader ethical crisis, eroding trust in both digital health systems and key stakeholders, such as health professionals and public authorities. Furthermore, unauthorised system and data access, as well as unauthorised data storage practices, undermine perceptions of security, making ethical violations persistent. In this respect, the study reveals that ethical breaches in digital health have a cumulative rather than linear effect on patient experience, and that the erosion of trust is the inevitable consequence of this process. This loss of trust undermines the patient–nurse and patient‐physician relationships, which form the ethical foundation of care (American Nurses Association 2024; Buhr et al. 2025; International Council of Nurses 2023). On the other hand, although the national personal health record system in Türkiye is defined as a secure system centered on patient control (Birinci 2023), this study's findings demonstrate a significant gap between design claims and perceived ethical security. When these experiences are combined with the erosion of procedural values, such as lack of transparency, ambiguous accountability, and limited data control, patients perceive digital health systems as ethically fragile, unpredictable, and uncontrollable. A previous study also emphasises that trust in digital health is not limited to data security alone; it must be considered alongside procedural values such as transparency, accountability, data ownership, and individual control (Vayena et al. 2018). In this context, the study reveals that ethical concerns in digital health constitute a holistic ethical governance problem that requires addressing privacy, autonomy, trust, and procedural values together, rather than focusing solely on technical security vulnerabilities.

### 4.3 When Ethical Violations Become Personal

Ethical issues in digital health systems generate strong emotional consequences. Ethical violations in digital health services lead to anger, vulnerability, loss of control, and various negative emotional responses, demonstrating that these violations are not limited to cognitive evaluations but also have a distinct emotional dimension. Houser et al. (Houser et al. 2023), in their systematic review of privacy risks in digital health, report that damage to patients' perception of private space negatively affects the sharing of sensitive health information and reduces emotional security. Similarly, misinterpretation of health data in digital systems or the detection of unauthorised changes can trigger anxiety and distrust (Aytekin et al. 2025; Buhr et al. 2025). Additionally, patients' access to their own health data without professional guidance, and their interpretation of this data alone, increases anxiety levels (Kyytsönen et al. 2024), which corresponds with the emotional reflections described in our study. The absence of sufficient control over the systems can deepen the emotional burden of ethical violations. In this context, evidence suggests that approaches based on artificial intelligence and machine learning may be used to identify psychological distress at an early stage (Johnson et al. 2025). Although these approaches do not eliminate the causes of ethical violations, they may help identify individuals experiencing psychological distress related to digital health systems at an early stage. These findings demonstrate that ethical violations in digital health create multidimensional and profound emotional effects on patient experience, and that emotional well‐being should be addressed as a central component in digital health ethics (Gybel Jensen et al. 2024; Nielsen et al. 2022).

### 4.4 Building an Ethically Trustworthy Digital Health System

Patients do not want to remain passive victims in the face of digital ethical violations; on the contrary, they ask for a transparent, traceable, accountable, controllable, and patient‐centered digital health system. Their demands for transparent access records showing who accesses health data, and for security measures such as strong authentication and encryption, are consistent with the literature (Pokharel and Kathayat 2025). It is emphasised that informed consent and clarity regarding data usage processes are indispensable for patient trust and conditional support, particularly in the context of secondary data use (Houser et al. 2023; Nebeker et al. 2021; Poulsen et al. 2025). Therefore, trust in digital health can be built not through isolated technical measures but through the combined operation of consent mechanisms, effective oversight structures, and accountability processes that enable public scrutiny (Vayena et al. 2018). Indeed, while WHO highlights the potential of digital health systems to increase effectiveness in patient care, it emphasises that if these systems are not supported by principles of transparency, traceability, and accountability, they may lead to ethical and governance problems (World Health Organization 2021a). For this reason, WHO highlights the prioritisation of robust governance frameworks, regular monitoring and evaluation mechanisms, and accountable policy tools in digital health strategies as a fundamental requirement (World Health Organization 2021a).

Participants' expectations for effective in‐system complaint mechanisms, strengthened oversight processes, and active involvement of patient rights units indicate that digital ethical violations need to be addressed at institutional and structural levels rather than at the individual level. Demands for sharing data access reports, rapid execution of data correction and deletion operations, and clear, understandable objection and application processes show that patients are adopting a strategic approach to regaining autonomy over their data. These expectations align with the core objectives of regulations such as KVKK (Kişisel Verilerin Korunması Kanunu), GDPR (General Data Protection Regulation), and HIPAA (Health Insurance Portability and Accountability Act), namely data privacy, security, and accountability principles (European Union 2016; Türkiye Cumhuriyeti 2016; U.S. Department of Health, and Human Services, Office for Civil Rights 2000). Strengthening oversight processes and visibly integrating patient rights into digital systems emerges as a critical element in building ethical trust.

However, participant experiences show that patients' interaction with health systems is increasingly shaped by their digital health literacy levels. Although digital tools offer potential benefits such as efficiency, accessibility, and personalisation, these benefits cannot be equally realised by individuals with limited digital competencies (Jawad 2024; Richardson et al. 2022). These findings indicate that digital health literacy should be addressed not only as an individual skill but also as part of the ethical responsibility of health systems. Results suggest that unless supported by inclusive design, alternative access routes, and supportive mechanisms, digital systems can undermine patient autonomy and equal treatment. Recent evidence shows that, when it comes to developing digital health literacy, simply teaching individuals how to use technology is not enough; a combined approach—including user‐centered co‐design, simple and accessible user interfaces, culturally adapted educational content, and ongoing technical and educational support provided by healthcare professionals—is more effective. Furthermore, tailoring digital health systems to individuals' needs strengthens users' sense of trust, control, and active participation in the system, thereby contributing to the reduction of digital inequalities (Barbati et al. 2025; Causio et al. 2025; Kilfoy et al. 2024). In this context, interventions aimed at improving digital health literacy should include not only patient education but also the redesign of the digital health ecosystem to be sensitive to users' information, skill, and ethical needs. Furthermore, previous studies have shown that such interventions increase patient empowerment, participation in digital health services, and trust in digital health systems (Alshanqiti et al. 2026).

### 4.5 Implications for Nursing Practice

The findings of this study have revealed that patients experience significant ethical concerns—particularly regarding data privacy, data autonomy, transparency, informed consent, and trust in digital health systems—when using digital health systems. These needs suggest that nurses—who maintain the closest and most continuous communication with patients—may play a significant role in supporting the ethical use of digital health technologies. In this context, nurses can be viewed as an “ethical interface” between patients and the digital health system—helping to develop digital health literacy, raise awareness about data rights, identify ethical concerns early on, encourage patients' active participation in decisions regarding their personal health data, and support ethical decision‐making processes.

In terms of future research, this study provides important qualitative evidence regarding patients' ethical experiences within digital health systems. However, it is recommended that future research be conducted with more diverse samples that include different age groups, educational levels, individuals with chronic conditions, and various healthcare settings. Furthermore, examining the relationships between digital health literacy, ethical awareness, patient trust, and the outcomes of using such technologies through mixed‐methods and longitudinal studies will contribute to a more comprehensive understanding of the short‐ and long‐term effects of ethical violations on the patient experience. Furthermore, there is a need to evaluate the effectiveness of intervention studies aimed at developing ethically safe digital health systems and patient‐centred digital health applications.

### 4.6 Strengths and Limitations

This study makes an original contribution as one of the first to examine patients' perceptions of digital ethical violations they encounter in digital health systems using qualitative methods. At a time when digital health applications are becoming increasingly widespread, focusing on the perceptions and experiences of patients who directly interact with the system is a key strength of the study. This approach provides a valuable perspective that places the patient at the centre of digital ethics discussions.

However, the study has several methodological limitations. This study was conducted with 13 patients receiving outpatient care at a single center. Findings should be assessed within the framework of analytical and contextual transferability in similar contexts. The reliance on interview data may have introduced social desirability bias or unintentionally omitted some experiences. Additionally, most participants were highly educated, which may have influenced their perceptions and ethical assessments of digital health technologies. Moreover, this educational homogeneity may have influenced participants' perceptions, digital literacy, and ethical evaluations of digital health technologies. Individuals with lower educational attainment may experience digital health systems differently and may express different concerns regarding usability, accessibility, privacy, and trust. Therefore, caution should be exercised when transferring these findings to populations with more diverse educational backgrounds.

Furthermore, this study was designed to be reported in accordance with the COREQ checklist to enhance transparency in qualitative research reporting (Tong et al. 2007). However, recent debates regarding qualitative research methodology suggest that COREQ may not fully reflect the epistemological diversity and interpretive nature of qualitative research, and that it may encourage the evaluation of research quality through checklists (Buus et al. 2026). For this reason, in this study, COREQ was used solely as a guide to support the reporting process; the study's trustworthiness and methodological rigour were addressed within the framework of interpretive analysis, supported by the principles of the phenomenological approach, researcher reflexivity, a detailed analysis process, and participant quotations.

## 5 Conclusion

The use of digital health technologies offers speed, practicality, and time savings in accessing health services, while also exposing the structural and ethical issues that accompany these benefits. From an ethical perspective, breaches of privacy, erosion of autonomy over health data, loss of trust in both digital systems and stakeholders, risks to data security, and procedural values within the systems (such as transparency, traceability, and accountability) emerge as significant fundamental problems. These ethical breaches affect not only cognitive understanding but also patients' emotional experiences, deepening their sense of loss of control and power. The inability to monitor who uses health data, for what purposes, and to what extent, along with the limited scope of interventions such as correcting, deleting, or restricting the sharing of data, leads patients to feel passive in relation to digital health systems.

In this context, preventing ethical violations and rebuilding patient trust are closely linked to effective health data management, the establishment of accountable and traceable systems, and the implementation of transparent access mechanisms. Strengthening informed consent processes and digital literacy initiatives that enable patients to make informed use of such technologies are fundamental components of ethical security. In conclusion, a sustainable and patient‐centred ethical framework in digital health systems is achievable not only through technical infrastructure but also through a holistic approach that strengthens autonomy, considers emotional impacts, and is based on trust. Furthermore, the findings suggest that nurses may serve as the ethical interface between patients and technology in digital health systems. Therefore, increasing nurses' awareness of digital ethics and supporting their active role in the early detection and reporting of ethical violations, as well as in patient empowerment processes, should be encouraged.

## Author Contributions

**Aysun Bayram:** conceptualization, data curation, formal analysis, investigation, methodology, project administration, resources, validation, supervision, visualization, writing – original draft, writing – review and editing. **Betül Bal:** conceptualization, data curation, formal analysis, investigation, methodology, project administration, resources, supervision, validation, writing – original draft, visualization. **Alvisa Palese:** conceptualization, formal analysis, methodology, supervision, writing – review and editing.

## Funding

The authors have nothing to report.

## Disclosure

No AI tool is generated.

## Ethics Statement

Ethics committee approval was obtained from the Yozgat Bozok University Ethics Committee for Social and Humanities Sciences (Date: 21.01.2026, Approval No: 1/4), and institutional permission was obtained from Karadeniz Technical University Farabi Hospital (Date: 04.02.2026, Number: E‐48814514‐299‐115292). We confirm that we have adhered to established ethical principles throughout this research.

## Consent

The purpose of the study, data collection procedures, confidentiality measures, and participants' right to withdraw from the study at any time without providing a reason were explained to the patients who agreed to participate. Written and verbal informed consent was obtained before data collection and audio recording commenced. All audio recordings were stored in an encrypted file accessible only to researchers authorised to access the data.

## Conflicts of Interest

The authors declare no conflicts of interest.

## Supporting information


**Table S1:** Consolidated criteria for reporting qualitative research (COREQ) guideline (Tong et al. 2007).

## Data Availability

The data underlying this study are not publicly available due to privacy and ethical restrictions.
