# Supplementary material for: Understanding Patients' Experiences of Digital Ethics in a Digitalised Healthcare System
Source: Nurs Open. 2026 Sep 4;13(9):e70736. doi: 10.1002/nop2.70736 (PMC13545412; doi:10.1002/nop2.70736)
Supplement: Supplementary file 1 [file NOP2-13-e70736-s001.docx]

**Supplementary Table 1.** Consolidated criteria for reporting qualitative research (COREQ) guideline (Tong et al. 2007)

| **No Item** | **Guide questions/description** | **In the study** |
| --- | --- | --- |
| ***Domain 1: Research team and reflexivity*** | | |
| *Personal Characteristics* | | |
| 1. Interviewer/facilitator | Which author conducted the interview? | Methods  *Data collection* |
| 2. Credentials | What were the researcher’s credentials? E.g. PhD, MD | Methods  *Data collection* |
| 3. Occupation | What was their occupation at the time of the study? | Methods  *Data collection* |
| 4. Gender | Was the researcher male or female? | Methods  *Data collection* |
| 5. Experience and training | What experience or training did the researcher have? | Methods  *Data collection* |
| *Relationship with participants* | | |
| 6. Relationship established | Was a relationship established prior to study commencement? | Methods  *Participants* |
| 7. Participant knowledge of the interviewer | What did the participants know about the researcher? e.g. personal goals, reasons for doing the research | Methods  *Data collection* |
| 8. Interviewer characteristics | What characteristics were reported about the interviewer/facilitator? e.g. Bias, assumptions, reasons and interests in the research topic | Methods  *Data collection and data analysis* |
| ***Domain 2: study design*** | | |
| *Theoretical framework* | | |
| 9. Methodological orientation and Theory | What methodological orientation was stated to underpin the study? e.g. grounded theory, discourse analysis, ethnography, phenomenology, content analysis | Methods  *Study design* |
| *Participant selection* | | |
| 10. Sampling | How were participants selected? e.g. purposive, convenience, consecutive, snowball | Methods  *Participants* |
| 11. Method of approach | How were participants approached? e.g. face-to-face, telephone, mail, email | Methods  *Participants* |
| 12. Sample size | How many participants were in the study? | Findings  *Participants* |
| 13. Non-participation | How many people refused to participate or dropped out? Reasons? | Findings  *Participants* |
| *Setting* | | |
| 14. Setting of data collection | Where was the data collected? e.g. home, clinic, workplace | Methods  *Participants* |
| 15. Presence of non-participants | Was anyone else present besides the participants and researchers? | Methods  *Data collection* |
| 16. Description of sample | What are the important characteristics of the sample? e.g. demographic data, date | Results  *Table 2* |
| *Data collection* | | |
| 17. Interview guide | Were questions, prompts, guides provided by the authors? Was it pilot tested? | Methods  *Data collection*  *Table 1* |
| 18. Repeat interviews | Were repeat interviews carried out? If yes, how many? | NR |
| 19. Audio/visual recording | Did the research use audio or visual recording to collect the data? | Methods  *Data collection* |
| 20. Field notes | Were field notes made during and/or after the interview or focus group? | Methods  *Data analysis* |
| 21. Duration | What was the duration of the interviews or focus group? | Methods  *Data collection* |
| 22. Data saturation | Was data saturation discussed? | Methods  *Participants and Data collection* |
| 23. Transcripts returned | Were transcripts returned to participants for comment and/or correction? | Methods  *Data analysis and Trustworthiness* |
| ***Domain 3: analysis and findings*** | | |
| *Data analysis* | | |
| 24. Number of data coders | How many data coders coded the data? | Methods  *Data analysis* |
| 25. Description of the coding tree | Did authors provide a description of the coding tree? | Methods  Table 3  Figure 1 |
| 26. Derivation of themes | Were themes identified in advance or derived from the data? | Methods  *Data analysis* |
| 27. Software | What software, if applicable, was used to manage the data? | NR |
| 28. Participant checking | Did participants provide feedback on the findings? | Methods  *Trustworthiness* |
| *Reporting* | | |
| 29. Quotations presented | Were participant quotations presented to illustrate the themes / findings? Was each quotation identified? e.g. participant number | Findings |
| 30. Data and findings consistent | Was there consistency between the data presented and the findings? | Findings |
| 31. Clarity of major themes | Were major themes clearly presented in the findings? | Findings |
| 32. Clarity of minor themes | Is there a description of diverse cases or discussion of minor themes? | Findings and Limitations |

**Legend.** NR, Not reported.

A. Tong, P. Sainsbury, and J. Craig, “Consolidated criteria for reporting qualitative research (COREQ): A 32-item checklist for interviews and focus groups,” International Journal for Quality in Health Care 19(2007): 349–357. <https://doi.org/10.1093/intqhc/mzm042>
